# T Cells, Interleukin-2 and Systemic Lupus Erythematosus—From Pathophysiology to Therapy

**DOI:** 10.3390/cells11060980

**Published:** 2022-03-12

**Authors:** Anselm Mak

**Affiliations:** 1 Department of Medicine, Yong Loo Lin School of Medicine, National University of Singapore, 10 Medical Dr, Singapore 117597, Singapore; mdcam@nus.edu.sg; 2 Division of Rheumatology, University Medicine Cluster, National University Health System, 1E Kent Ridge Road, Level 10, Singapore 119228, Singapore

**Keywords:** SLE, lupus, T cell, regulatory, interleukin-2

## Abstract

The phenotypic and functional complexities of T cells engender complicated and often confusing concepts as to how T cells ignite, accelerate and brake the inflammatory processes involved in systemic lupus erythematosus (SLE), let alone the plasticity of T cells that takes place under different immunological contexts. Nevertheless, being one of the prime survival factors of T cells, interleukin (IL)-2 plays a potentially critical role in many immunological scenarios during the pathophysiological process of SLE. Here, the pathophysiology of lupus T cells and current, as well as ongoing, therapeutic approaches of SLE that involve low-dose IL-2 administration will be highlighted. The mechanisms of IL-2 deficiency in SLE pathophysiology, the effects of low-dose IL-2 on T cells and restoration of lupus manifestations in murine SLE models, as well as the efficacy and safety of clinical trials that evaluated low-dose IL-2-containing regimens in patients with SLE will be discussed.

## 1. Introduction

The pathophysiology of systemic lupus erythematosus (SLE) is complex. The involvement of innate and adaptive immunity in the initiation and perpetuation of the disease course of SLE implies that the interplay between various leucocyte subsets, cytokines, chemokines and resident tissue cells are pivotal to the pathophysiology of the condition [1]. Over the past 2 decades, the roles that T cells are involved in the pathophysiological process of SLE has been increasingly recognized [2]. The production of a wide array of SLE-related autoantibodies by B cells and antibody-forming cells (AFCs) obviously signifies the substantial role played by T cells in SLE because crucial processes that take place in B cells including proliferation and production, class-switching and affinity maturation of antibodies require their intimate interactions with T cells in the germinal centers (GC) [3,4]. Furthermore, the breach of immune tolerance, particularly that of peripheral immune tolerance, is postulated to be one of the main mechanisms that initiates and perpetuates the pathological processes of SLE [5]. In this regard, the regulatory function of T cells that is designed for suppression of autoreactive B and T cells, is compromised in SLE [6].

Although successful responses to B cell depletion therapy (BCDT) in patients with SLE have been reported in case series [7] and improvement of serological abnormalities of lupus has often been observed after BCDT [8], the failure of reaching pre-defined primary endpoints in major clinical trials that evaluated the efficacy of BCDT in the treatment of SLE [8,9] implies that targeting B cells *per se* may be insufficient to curb the complicated autoimmune condition. Because T cells are largely indispensable in enhancing the pathological activity of B cells in SLE, pharmacological interventions of T cells intuitively offer an alternative or an additional therapeutic strategy that can potentially control the disease activity of SLE. In fact, drugs that suppress T cell activation, typically the calcineurin inhibitors, have been found efficacious in reducing SLE disease activity, particularly in lupus glomerulonephritis (LN) [10,11]. Nevertheless, suppressing T cells alone may not be ideal in dealing with SLE either. Relapse of nephrotic syndrome after cessation of cyclosporine [12] and the non-inferiority of mycophenolate mofetil versus tacrolimus in the treatment of LN [10] are representative of the limitation of targeting T cells *per se* in the treatment of SLE. Given the complexity of the phenotypic and functional diversity, as well as the plasticity of T cells, the art and science as to how T cells should be manipulated in treating individual patients with SLE remain to be explored.

Interleukin (IL)-2 is one of the prime cytokines that activates and maintains the survival of T cells [13]. Being apparently counterintuitive, IL-2 deficiency has been observed in many autoimmune conditions such as non-obese diabetic (NOD) mice [14], and in patients with rheumatoid arthritis (RA) [15], type-1 diabetes mellitus (T1DM) [16] and SLE [17]. In murine models, low-dose IL-2 therapy was found to be effective in the treatment of NOD mice [18] and female NZB/W F_1_ lupus-prone mice [19]. Beyond animal models, clinical studies have found that low-dose human-recombinant IL-2 treatment was safe in immunosuppressing patients with hepatitis C-induced vasculitis (HCV vasculitis) [20], chronic graft-versus-host disease (GvHD) [21] and SLE [22]. In this mini-review, the role played by IL-2 in autoimmunity, particularly its mechanistic actions on follicular helper T cells (T_FH_) and regulatory T cells (T_reg_), will be critically discussed. The potential mechanisms of how restoration of IL-2 level mitigates the clinical manifestations of SLE, and the current evidence of the efficacy and safety of low-dose IL-2 therapy in patients with SLE will be reviewed.

## 2. Abnormalities of Lupus T Cells

T cell receptors (TCR) physically and functionally associate with CD3, which enhances clonal differentiation and maturation of T cells upon engagement with membrane-bound major histocompatibility complex (MHC) molecules. MHC molecules are expressed on antigen-presenting cells (APCs) that present specific antigen sequences to T cells [23]. The majority (95%) of TCRs consist of membrane-bound α and β chains (αβTCR), which extend short cytoplasmic tails essential for downstream intracellular signaling. The minority of TCRs comprise a γ chain and a δ chain (γδTCR) that are expressed on some populations of T cells residing on mucosal and epithelial surfaces [24]. The TCR-CD3 complex initiates signal transduction upon TCR engagement with MHC molecules. CD3 consists of four invariant polypeptides (γ, δ, ε, ζ). The TCR-CD3 complex is arranged in such a fashion that the two TCR chains (two positively charged α/β or γ/δ chains) associate with 2ε, 2ζ, 1γ and one δ polypeptide chain, which are negatively charged, enhancing electrostatic stabilization of the complex [24]. The cytoplasmic portions of the ζ and η chains are important in TCR signal transduction for their crucial anatomical and functional associations with the immunoreceptor tyrosine-based activation motifs (ITAMs) [24]. ITAMs are phosphorylation targets by various protein kinases that further amplify intracellular signaling cascades. In normal situations, the ζ-associated protein 70 (ZAP-70) pathway is preferentially activated in a few minutes following TCR stimulation, leading to T cell activation [24].

Besides the CD3-TCR complex, FcγR also functionally associates with the ITAMs in T cells. Instead of activating the ZAP-70 pathway when the CD3ζ chain is engaged, engagement with FcγR triggers signaling through the spleen tyrosine kinase (Syk) pathway when TCR is activated [25]. In lupus T cells, reduced stability, synthesis and expression, and enhanced degradation of CD3ζ are consistently evident [26]. The resultant quantitative and functional deficiencies of CD3ζ in lupus T cells lead to a reciprocal increase in the expression of FcγR, enhancing pathway “rewiring” towards Syk stimulation, and consequential higher calcium influx into T cells compared to that triggered by the CD3ζ-ZAP-70 pathway [26]. Such aberrant signal rewiring in lupus T cells gives rise to stronger phosphorylation signals that lowers the TCR activation threshold and intensifies TCR-derived signals, contributing to T cell hyperactivation [27]. Additionally, the increase in intracellular serine/threonine protein phosphatase 2A (PP2A) activity in lupus T cells contributes to aberrant TCR signaling due to the utilization of the FcεRIγ chain instead of the native CD3ζ chain via Elf-1 dephosphorylation [27,28], diverting to another re-wiring mechanism that triggers higher calcium influx into lupus T cells, which activates cytoplasmic calcineurin. Calcineurin dephosphorylates the inactive form of cytosolic nuclear factor of activated T cells (NF-ATc2), rendering it active and facilitating its translocation into the nucleus, amplifying CD40L and CD70 gene expression of T cells by binding with their respective upstream promoters [2]. Increased expressions of the costimulatory CD40L and CD70 molecules on T cells promote differentiation and proliferation of B cells by interacting with CD40 and CD27, which are expressed on the latter. Under the concurrent action of IL-10 produced by various leucocyte types including T cells, monocytes, macrophages and dendritic cells, and IL-21, which is expressed mainly in follicular T helper cells in GC, class switching and somatic hypermutation of immunoglobulins are substantially enhanced, leading to the production of high-affinity autoantibodies [29,30].

The lower threshold of lupus T cell activation is also partly due to pre-aggregated lipid rafts on T cell surfaces [31]. Lipid rafts are lipid-rich micro-domains on the T cell plasma membrane that consist of cholesterol, sphingomyelin, and glycosphingolipids where TCR and relevant signaling molecules physically and functionally aggregate [31]. During the inactivated state, lipid rafts are evenly distributed throughout the cell membrane but in lupus T cells, lipid rafts are clustered despite minimal stimulation [32,33]. Clustering of lipid rafts brings relevant signal transduction molecules in close physical proximity that lowers the threshold of TCR activation and tremendously enhances the intensity of intracellular signaling [32,33].

Aberrant and amplified TCR signaling, lipid raft formation and increased costimulatory molecule expression render lupus T cells metabolically active in a persistent fashion [34]. The flexibility of metabolic shift and the plasticity of T cells endow them with the ability to adapt to different phenotypes and functions based on the metabolic and functional demands. For instance, enhanced glycolysis and the pentose phosphate pathway activate Th17 cells, while fatty acid oxidation and oxidative phosphorylation are crucial in energy supply to enhance T_reg_ function [34].

## 3. Physiology and Action of IL-2

IL-2, a pleiotropic cytokine produced mostly by conventional T cells upon TCR and co-stimulatory molecule engagement, is a crucial factor for T cell survival [35]. IL-2 is a member of the common γ-chain family that signals through the IL-2 receptor (IL-2R) of two conformations. The high affinity IL-2 receptor (IL-2R) consists of an α (CD25), β (CD122) and a common γ chain (CD132) that form a heterotrimer [36]. Although the affinity between IL-2 and CD25 is weak, their interaction induces a conformational change in IL-2 that enhances its stabilization and binding affinity to the β chain of the IL-2R [37]. Activation of IL-2R activates phosphorylation of Janus-Activated Kinase (JAK) 1 and JAK3 and initiates downstream signaling cascades that activate MAPK and PI-3K pathways and subsequent activation and translocation of STAT5 into the nucleus [38]. While activation of TCR induces transient upregulation of CD25 that forms high-affinity IL-2R in memory and naïve T cells, CD25 is highly and persistently expressed on FoxP3-expressing CD4^+^ T_reg_, endowing them with an advantage to compete for available IL-2 in the circulation and microenvironment [39]. In response to IL-2, the binding of STAT5 to the *FoxP3* locus promotes FoxP3 expression, a pivotal regulator of T_reg_ differentiation [40]. Of note, T_reg_ essentially cannot express IL-2 because of the repressive effect of FoxP3. Therefore, the source of IL-2 for T_reg_ is chiefly exogenous [35,41]. Based on these observations, differential expressions of CD25 in different T cell subsets appear to determine their responses to IL-2. While a high IL-2 concentration activates the majority of T cell subsets including the T_reg_, effector T cells and natural killer (NK) cells, T_reg_ that are highly CD25 expressing are preferentially activated in response to the context of low IL-2 environment.

## 4. IL-2 Deficiency and Hypo-Responsiveness in SLE—Potential Mechanisms and Impact

Instead of immunodeficiency, deficiency of IL-2 is observed in many autoimmune conditions, both in animal models and human autoimmune diseases. Notably, both IL-2 and IL-2R-deficient mice develop life-threatening autoimmunity [42,43]. T cells isolated from non-obese diabetic (NOD) mice were found to produce less IL-2 [14], and similar has been observed in patients with RA, T1DM and SLE [15,16,17,44]. The mechanisms that lead to low IL-2 production in autoimmune conditions including SLE, is not fully understood. Alterations of the expression of regulatory elements that mediate *IL-2* transcription such as NF-κB, NFAT-c2, PP2A, microRNAs (e.g., miR-200a-3p) and phosphorylated cAMP-responsive element modulator (p-CREM) are evident in patients with SLE [45,46,47,48]. Furthermore, IL-23, a cytokine that was found to be elevated in patients with SLE and associated with active SLE [49], has been shown to upregulate IL-7 but suppress IL-2 production [49]. When lupus T cells were co-cultured with IL-23, expansion of double-negative T cells (DNTC) and T_FH_ were observed [49]. In addition, MRL/lpr lupus-prone mice knocked out for IL-23 receptors (IL-23R) revealed less severe LN, which was mechanistically explained by the restoration of IL-2 and a decrease in IL-17 production by T cells [49], together with reduced T_FH_ and APCs, and reduced anti-dsDNA levels. Similarly, B6.lpr^−/−^ lupus-prone mice devoid of IL-23R abrogated the development of LN, coupled with a reduction of the number of DNTC, IL-17A-producing cells in lymph nodes and anti-dsDNA antibody production [50]. Along with low IL-2 production, hypo-responsiveness to IL-2 in lupus T cells was evident [51]. It has been postulated that high serum IL-6 level and increased IL-6 signaling that occur in patients with SLE might be associated with reduced expression of IL-2R [51,52]. In addition, increased soluble form of IL-2R in patients with SLE potentially competes with membrane-bound CD25 for IL-2, rendering lupus T cells less responsive to IL-2 [53].

In the GC, T_FH_ closely communicates with and activates follicular B cells for the production of high-affinity autoantibodies. B-cell lymphoma 6 protein (BCL6) is a master regulatory factor for differentiation of naïve T cells to T_FH_ [54]. IL-2 signaling potently inhibits BCL6 expression and mitigates T_FH_ differentiation [55]. Therefore, the low IL-2 environment in SLE favors the development of T_FH_, leading to the production of high-affinity autoantibodies that are associated with various clinical manifestations of SLE.

**Figure 1:**
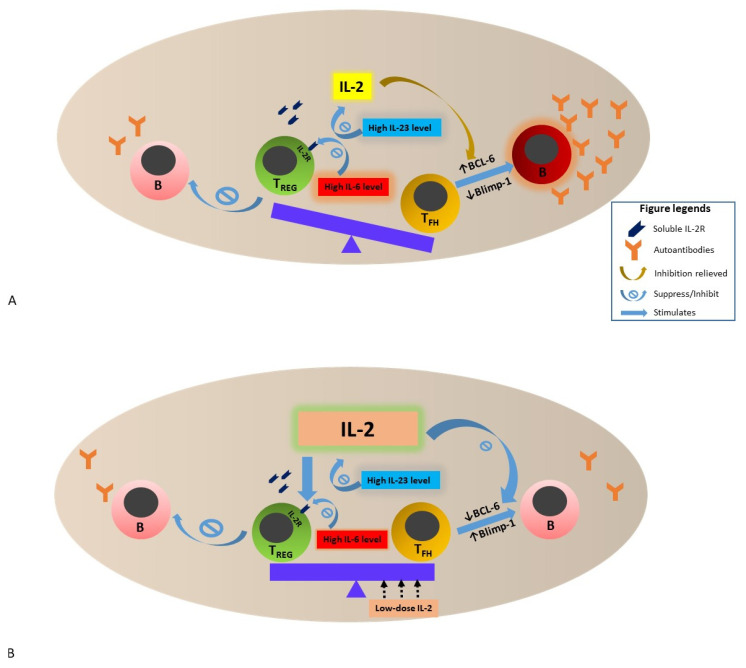
Schematic presentation of the interactions among T_FH_, T_REG_, IL-2, IL-2R and B cells in a lymph node (or a second lymphoid organ) in the setting of SLE. (**A**). The reasons for low IL-2 environment in SLE are multifactorial—reduction of IL-2 transcription in T cells, increase in soluble IL-2R that bind to IL-2 (see text) and increase in IL-23 expressions are among some of the mechanisms proposed. Furthermore, high IL-6 expression in active SLE reduces the expression of IL-2 receptors expressed on T_REG_, leading to reduction of _TREG_ regulatory function—for example, suppression of B cells to produce autoantibodies. These alterations impact peripheral immune tolerance by enhancing the development and differentiation of T_FH_ while suppressing T_REG_ function. Coupled with the lifted inhibition of BCL-6 expression and suppression of Blimp-1 expression by reduced IL-2 signaling, proliferation and function of T_FH_ are fueled, leading to activation of B cells and antibody-forming cells and subsequent increased production of high-affinity autoreactive antibodies in SLE. (**B**). Low-dose IL-2 therapy restores the physiologically balanced activity between T_REG_ and T_FH_ by reversing the low IL-2 environment and stimulating T_REG_ activity via direct binding of IL-2 to IL-2R of T_REG_. The higher IL-2 microenvironment also restores the balance between BCL-6 and Blimp-1 expression, leading to reduction in autoantibody formation. The impact of low-dose IL-2 therapy on IL-6 and IL-23 in SLE is still unclear. Abbreviations: SLE, systemic lupus erythematosus; IL, interleukin; T_REG_, regulatory T cell; T_FH_, follicular T-helper cell; IL-2R, IL-2 receptor; B, B cells; BCL-6; B-cell lymphoma 6 protein; Blimp-1, B lymphocyte-induced maturation protein-1.

Taken together, the low IL-2 environment in SLE that is potentially related to reduced IL-2 transcription in T cells, high soluble CD25, and high serum IL-6 and IL-23 levels impact peripheral immune tolerance by enhancing the development and differentiation of T_FH_, and suppressing T_reg_ function. Further, IL-2 shortage and reduced IL-2 signaling lift the inhibition of BCL6 expression and suppress Blimp-1 expression that negatively regulates BCL6 expression. These immune alterations collectively fuel T_FH_ development and differentiation that activate B cells and APCs, with subsequent production of high-affinity autoreactive antibodies in SLE (see Figure 1).

## 5. Restoration of IL-2 in Murine Lupus Model

Alleviation of lupus manifestations was achieved in lupus-prone murine models by reversing the low IL-2 environment. In NZB/NZW F_1_ lupus-prone mice, IL-2 deficiency was shown to induce hyperactivity of conventional T cells, and low T_reg_ to conventional T cell ratio [56]. Low-dose IL-2 treatment in these mice restored T_reg_ homeostasis, as evidenced by an increase in T_reg_ numbers in the peripheral circulation, lymphoid organs and kidneys, leading to reduction in renal inflammation and prolonged survival of these mice [56,57]. In another study using the same mouse model, the use of a complex consisting of IL-2 and anti-IL2 monoclonal antibody (JES6-1) demonstrated attenuation of renal glomerular and tubular injury, vasculitis and renal depositions of C3 and anti-dsDNA antibodies, coupled with improvement of peripheral disease activity including elevation of serum complements and reduction in anti-dsDNA level by sustained expansion of CD4^+^CD25^+^FoxP3^+^ T_reg_ [58]. Treatment of MRL/lpr lupus-prone mice with IL-2-recombinant adeno-associated virus led to amelioration of lupus-related skin, lung and renal pathological lesions, accompanied by a significant reduction of IL-17-producing DNTC and increase in T_reg_ number [59].

## 6. Clinical Observation and Therapeutic Trials of Low-Dose IL-2 Therapy in Patients with SLE

Low-dose IL-2 preferentially activates T_reg_ due to their heightened expression of CD25 [51]. After the first report of the successful use of low-dose IL-2 in a 36-year-old SLE patient as evidenced by the subsidence of arthritis and skin eruption as well as serological improvement and functional improvement of CD25^++^FoxP3^+^CD127^lo^ T_reg_ cells [60], a series of 5 patients with refractory SLE who received daily subcutaneous injections of 1.5 million IU of human IL-2 (aldesleukin) for five consecutive days were found to have selective expansion of T_reg_ number despite the absence of formal clinical assessment of SLE disease activity [61]. In a prospective open-label study of 50 patients with refractory SLE who received low-dose IL-2 for 3 to 5 days monthly and 0.5 mg rapamycin (oral) alternate days, significant improvement of SLE disease activity was noted up to 24 weeks, accompanied by a decrease of circulating Th17/T_reg_ ratio [62]. Subsequently, two small clinical trials that reported the efficacy and safety of low-dose IL-2 use in patients with SLE have been published [63,64]. The first trial is a prospective, open-labelled study that evaluated the safety and efficacy of 3 three cycles of subcutaneous low-dose recombinant human IL-2 (rhIL-2) given at a dose of 1 million IU alternate days for 2 weeks followed by a break of 2 weeks. Over 89% of the 38 patients with active SLE who completed the study achieved Systemic lupus erythematosus Response Index (SRI)-4 response at the end of 12 weeks, accompanied by significant reductions in the SELENA-SLEDAI and reduction of over 50% of glucocorticoid dose compared with baseline in over two-third of the patients [63]. In a subsequent randomized, placebo-controlled trial of 60 patients with active SLE by the same group [64], the same IL-2 treatment regime as in the open-labelled study [63] resulted in 55.17% SRI-4 response in the IL-2 group compared with 30% in the placebo group at week 12. Although SRI-4 response did not statistically differ between both groups at week 12, the SRI-4 response rate of the IL-2 group was significantly higher than that of the placebo group at week 24 [64]. In addition, 53.85% of patients in the IL-2 group had complete remission of LN compared with 16.67% in the placebo group. No serious infection and adverse events were reported in both trials [63,64]. Further in vitro studies of samples from the two trials demonstrated that low-dose IL-2 treatment led to qualitative and functional improvement of T_reg_ [63,64], one study revealed enhanced NK cells [63] and the other demonstrated a significant fall in the T_FH_^+^Th17/T_reg_ cell ratio [64]. Another two phase II clinical trials are currently underway to further address the safety and efficacy of IL-2 use in patients with SLE. One of them is an open-labelled phase II study that was completed in December 2019 with 16 subjects recruited (Charact-IL-2: NCT03312335). The other study (NCT01988506) was completed on April 2021, with 81 participants with various autoimmune diseases studied. Results of these two studies are being awaited.

## 7. Conclusions and Perspectives

T cells are instrumental in mediating the immunopathophysiology of SLE. The many faces and functions of T cells make therapeutic strategies that target T cells technically complicated. Despite conflicting data regarding the number and function of T_reg_ in patients with SLE, promising clinical responses were observed in patients with SLE who received low-dose IL-2 therapy. In addition, as a critical cytokine that activates and maintains the survival of T cells [13], low-dose IL-2 therapy unlikely leads to major infective complications, in contrast to various anti-cytokine therapies and BCDT that can potentially lead to severe immunosuppression [65,66]. Yet, two major challenges are anticipated pertaining to the use of low-dose IL-2 in the treatment of SLE. First, the short half-life of IL-2 implies that repeated injections in short durations are required. Second, IL-2 therapy alone might not be sufficient to control SLE effectively given the complexity of the pathophysiology of SLE that involves many cell types and the functional changes of certain cell types, particularly the T cells, in different immunological contexts. The use of IL-2 specific monoclonal antibody together with low-dose IL-2 is one of the strategies that lengthens the half-life of IL-2 [58], particularly those that increase IL-2 activity in CD25^+^ T cells, with an aim to selectively regulate T_reg_.

Common to most autoimmune conditions, multiple perturbations and pathways are likely involved in the pathophysiology of SLE [67]. Therefore, targeting different disease mechanisms concurrently or sequentially is theoretically more promising than manipulating a single target such as a disease pathway, cytokine, chemokine or cell type in complex autoimmune diseases such as SLE. Based on this principle, careful evaluation of the safety and efficacy of the combination of low-dose IL-2 with other biologic therapy such as BCDT (concurrent or sequential) can be considered as novel treatment strategies of SLE.

## References

[1] Tsokos G.C. (2011). Systemic lupus erythematosus. N. Engl. J. Med..

[2] Mak A., Kow N.Y. (2014). The pathology of T cells in systemic lupus erythematosus. J. Immunol. Res..

[3] Kennedy D.E., Clark M.R. (2021). Compartments and Connections within the Germinal Center. Front. Immunol..

[4] Reijm S., Kissel T., Toes R.E.M. (2020). Checkpoints controlling the induction of B cell mediated autoimmunity in human autoimmune diseases. Eur. J. Immunol..

[5] Kanta H., Mohan C. (2009). Three checkpoints in lupus development: Central tolerance in adaptive immunity, peripheral amplification by innate immunity and end-organ inflammation. Genes Immun..

[6] Longhi M.S., Ma Y., Grant C.R., Samyn M., Gordon P., Mieli-Vergani G., Vergani D. (2013). T-regs in autoimmune hepatitis-systemic lupus erythematosus/mixed connective tissue disease overlap syndrome are functionally defective and display a Th1 cytokine profile. J. Autoimmun..

[7] Smith K.G., Jones R.B., Burns S.M., Jayne D.R. (2006). Long-term comparison of rituximab treatment for refractory systemic lupus erythematosus and vasculitis: Remission, relapse, and re-treatment. Arthritis Rheum..

[8] Rovin B.H., Furie R., Latinis K., Looney R.J., Fervenza F.C., Sanchez-Guerrero J., Maciuca R., Zhang D., Garg J.P., Brunetta P. (2012). Efficacy and safety of rituximab in patients with active proliferative lupus nephritis: The Lupus Nephritis Assessment with Rituximab study. Arthritis Rheum..

[9] Merrill J.T., Neuwelt C.M., Wallace D.J., Shanahan J.C., Latinis K.M., Oates J.C., Utset T.O., Gordon C., Isenberg D.A., Hsieh H.J. (2010). Efficacy and safety of rituximab in moderately-to-severely active systemic lupus erythematosus: The randomized, double-blind, phase II/III systemic lupus erythematosus evaluation of rituximab trial. Arthritis Rheum..

[10] Mok C.C., Ying K.Y., Yim C.W., Siu Y.P., Tong K.H., To C.H., Ng W.L. (2016). Tacrolimus versus mycophenolate mofetil for induction therapy of lupus nephritis: A randomised controlled trial and long-term follow-up. Ann. Rheum. Dis..

[11] Mok C.C., Ho L.Y., Ying S.K.Y., Leung M.C., To C.H., Ng W.L. (2020). Long-term outcome of a randomised controlled trial comparing tacrolimus with mycophenolate mofetil as induction therapy for active lupus nephritis. Ann. Rheum. Dis..

[12] Austin H.A., Illei G.G., Braun M.J., Balow J.E. (2009). Randomized, controlled trial of prednisone, cyclophosphamide, and cyclosporine in lupus membranous nephropathy. J. Am. Soc. Nephrol..

[13] Tahvildari M., Dana R. (2019). Low-Dose IL-2 Therapy in Transplantation, Autoimmunity, and Inflammatory Diseases. J. Immunol..

[14] Diaz-de-Durana Y., Lau J., Knee D., Filippi C., Londei M., McNamara P., Nasoff M., DiDonato M., Glynne R., Herman A.E. (2013). IL-2 immunotherapy reveals potential for innate beta cell regeneration in the non-obese diabetic mouse model of autoimmune diabetes. PLoS ONE.

[15] Kitas G.D., Salmon M., Farr M., Gaston J.S., Bacon P.A. (1988). Deficient interleukin 2 production in rheumatoid arthritis: Association with active disease and systemic complications. Clin. Exp. Immunol..

[16] Hulme M.A., Wasserfall C.H., Atkinson M.A., Brusko T.M. (2012). Central role for interleukin-2 in type 1 diabetes. Diabetes.

[17] Linker-Israeli M., Bakke A.C., Kitridou R.C., Gendler S., Gillis S., Horwitz D.A. (1983). Defective production of interleukin 1 and interleukin 2 in patients with systemic lupus erythematosus (SLE). J. Immunol..

[18] Nagy N., Kaber G., Kratochvil M.J., Kuipers H.F., Ruppert S.M., Yadava K., Yang J., Heilshorn S.C., Long S.A., Pugliese A. (2021). Weekly injection of IL-2 using an injectable hydrogel reduces autoimmune diabetes incidence in NOD mice. Diabetologia.

[19] Liang K., He J., Wei Y., Zeng Q., Gong D., Qin J., Ding H., Chen Z., Zhou P., Niu P. (2021). Sustained low-dose interleukin-2 therapy alleviates pathogenic humoral immunity via elevating the Tfr/Tfh ratio in lupus. Clin. Transl. Immunol..

[20] Saadoun D., Rosenzwajg M., Joly F., Six A., Carrat F., Thibault V., Sene D., Cacoub P., Klatzmann D. (2011). Regulatory T-cell responses to low-dose interleukin-2 in HCV-induced vasculitis. N. Engl. J. Med..

[21] Koreth J., Matsuoka K., Kim H.T., McDonough S.M., Bindra B., Alyea E.P., Armand P., Cutler C., Ho V.T., Treister N.S. (2011). Interleukin-2 and regulatory T cells in graft-versus-host disease. N. Engl. J. Med..

[22] Humrich J.Y., Riemekasten G. (2019). Low-dose interleukin-2 therapy for the treatment of systemic lupus erythematosus. Curr. Opin. Rheumatol..

[23] Kow N.Y., Mak A. (2013). Costimulatory pathways: Physiology and potential therapeutic manipulation in systemic lupus erythematosus. Clin. Dev. Immunol..

[24] Roth D.B., Male D., Brostoff J., Roth D.B., Roitt I.M. (2013). T Cell Receptors and MHC Molecules. Immunology.

[25] Krishnan S., Warke V.G., Nambiar M.P., Tsokos G.C., Farber D.L. (2003). The FcR gamma subunit and Syk kinase replace the CD3 zeta-chain and ZAP-70 kinase in the TCR signaling complex of human effector CD4 T cells. J. Immunol..

[26] Liossis S.N., Ding X.Z., Dennis G.J., Tsokos G.C. (1998). Altered pattern of TCR/CD3-mediated protein-tyrosyl phosphorylation in T cells from patients with systemic lupus erythematosus. Deficient expression of the T cell receptor zeta chain. J. Clin. Investig..

[27] Krishnan S., Farber D.L., Tsokos G.C. (2003). T cell rewiring in differentiation and disease. J. Immunol..

[28] Juang Y.T., Wang Y., Jiang G., Peng H.B., Ergin S., Finnell M., Magilavy A., Kyttaris V.C., Tsokos G.C. (2008). PP2A dephosphorylates Elf-1 and determines the expression of CD3zeta and FcRgamma in human systemic lupus erythematosus T cells. J. Immunol..

[29] Young C., Brink R. (2021). The unique biology of germinal center B cells. Immunity.

[30] Qiu H., Wu H., Chan V., Lau C.S., Lu Q. (2017). Transcriptional and epigenetic regulation of follicular T-helper cells and their role in autoimmunity. Autoimmunity.

[31] Huang S.C., Tsai H.F., Tzeng H.T., Liao H.J., Hsu P.N. (2011). Lipid raft assembly and Lck recruitment in TRAIL costimulation mediates NF-κB activation and T cell proliferation. J. Immunol..

[32] Jury E.C., Kabouridis P.S., Flores-Borja F., Mageed R.A., Isenberg D.A. (2004). Altered lipid raft-associated signaling and ganglioside expression in T lymphocytes from patients with systemic lupus erythematosus. J. Clin. Investig..

[33] Krishnan S., Nambiar M.P., Warke V.G., Fisher C.U., Mitchell J., Delaney N., Tsokos G.C. (2004). Alterations in lipid raft composition and dynamics contribute to abnormal T cell responses in systemic lupus erythematosus. J. Immunol..

[34] Sun L., Fu J., Zhou Y. (2017). Metabolism Controls the Balance of Th17/T-Regulatory Cells. Front. Immunol..

[35] Kolios A.G.A., Tsokos G.C., Klatzmann D. (2021). Interleukin-2 and regulatory T cells in rheumatic diseases. Nat. Rev. Rheumatol..

[36] Votavova P., Tomala J., Kovar M. (2014). Increasing the biological activity of IL-2 and IL-15 through complexing with anti-IL-2 mAbs and IL-15Rα-Fc chimera. Immunol. Lett..

[37] Wang X., Rickert M., Garcia K.C. (2005). Structure of the quaternary complex of interleukin-2 with its alpha, beta, and gammac receptors. Science.

[38] Ross S.H., Cantrell D.A. (2018). Signaling and Function of Interleukin-2 in T Lymphocytes. Annu. Rev. Immunol..

[39] Zhang R., Miao J., Zhu P. (2021). Regulatory T cell heterogeneity and therapy in autoimmune diseases. Autoimmun. Rev..

[40] Schwartz D.M., Kanno Y., Villarino A., Ward M., Gadina M., O’Shea J.J. (2017). JAK inhibition as a therapeutic strategy for immune and inflammatory diseases. Nat. Rev. Drug Discov..

[41] Zeiser R., Negrin R.S. (2008). Interleukin-2 receptor downstream events in regulatory T cells: Implications for the choice of immunosuppressive drug therapy. Cell Cycle.

[42] Malek T.R., Yu A., Vincek V., Scibelli P., Kong L. (2002). CD4 regulatory T cells prevent lethal autoimmunity in IL-2Rbeta-deficient mice. Implications for the nonredundant function of IL-2. Immunity.

[43] Sadlack B., Merz H., Schorle H., Schimpl A., Feller A.C., Horak I. (1993). Ulcerative colitis-like disease in mice with a disrupted interleukin-2 gene. Cell.

[44] Alcocer-Varela J., Alarcón-Segovia D. (1982). Decreased production of and response to interleukin-2 by cultured lymphocytes from patients with systemic lupus erythematosus. J. Clin. Investig..

[45] Katsuyama E., Yan M., Watanabe K.S., Narazaki M., Matsushima S., Yamamura Y., Hiramatsu S., Ohashi K., Watanabe H., Katsuyama T. (2017). Downregulation of miR-200a-3p, Targeting CtBP2 Complex, Is Involved in the Hypoproduction of IL-2 in Systemic Lupus Erythematosus-Derived T Cells. J. Immunol..

[46] Sharabi A., Kasper I.R., Tsokos G.C. (2018). The serine/threonine protein phosphatase 2A controls autoimmunity. Clin. Immunol..

[47] Solomou E.E., Juang Y.T., Gourley M.F., Kammer G.M., Tsokos G.C. (2001). Molecular basis of deficient IL-2 production in T cells from patients with systemic lupus erythematosus. J. Immunol..

[48] Rudensky A.Y., Gavin M., Zheng Y. (2006). FOXP3 and NFAT: Partners in tolerance. Cell.

[49] Dai H., He F., Tsokos G.C., Kyttaris V.C. (2017). IL-23 Limits the Production of IL-2 and Promotes Autoimmunity in Lupus. J. Immunol..

[50] Kyttaris V.C., Zhang Z., Kuchroo V.K., Oukka M., Tsokos G.C. (2010). Cutting edge: IL-23 receptor deficiency prevents the development of lupus nephritis in C57BL/6-lpr/lpr mice. J. Immunol..

[51] Ballesteros-Tato A., Papillion A. (2019). Mechanisms of action of low-dose IL-2 restoration therapies in SLE. Curr. Opin. Immunol..

[52] Papillion A., Powell M.D., Chisolm D.A., Bachus H., Fuller M.J., Weinmann A.S., Villarino A., O’Shea J.J., León B., Oestreich K.J. (2019). Inhibition of IL-2 responsiveness by IL-6 is required for the generation of GC-TFH cells. Sci. Immunol..

[53] Tokano Y., Murashima A., Takasaki Y., Hashimoto H., Okumura K., Hirose S. (1989). Relation between soluble interleukin 2 receptor and clinical findings in patients with systemic lupus erythematosus. Ann. Rheum. Dis..

[54] Nurieva R.I., Chung Y., Martinez G.J., Yang X.O., Tanaka S., Matskevitch T.D., Wang Y.H., Dong C. (2009). Bcl6 mediates the development of T follicular helper cells. Science.

[55] Mountz J.D., Hsu H.C., Ballesteros-Tato A. (2019). Dysregulation of T Follicular Helper Cells in Lupus. J. Immunol..

[56] Humrich J.Y., Morbach H., Undeutsch R., Enghard P., Rosenberger S., Weigert O., Kloke L., Heimann J., Gaber T., Brandenburg S. (2010). Homeostatic imbalance of regulatory and effector T cells due to IL-2 deprivation amplifies murine lupus. Proc. Natl. Acad. Sci. USA.

[57] Rose A., von Spee-Mayer C., Kloke L., Wu K., Kühl A., Enghard P., Burmester G.R., Riemekasten G., Humrich J.Y. (2019). IL-2 Therapy Diminishes Renal Inflammation and the Activity of Kidney-Infiltrating CD4+ T Cells in Murine Lupus Nephritis. Cells.

[58] Yan J.J., Lee J.G., Jang J.Y., Koo T.Y., Ahn C., Yang J. (2017). IL-2/anti-IL-2 complexes ameliorate lupus nephritis by expansion of CD4+ CD25+ Foxp3+ regulatory T cells. Kidney Int..

[59] Mizui M., Koga T., Lieberman L.A., Beltran J., Yoshida N., Johnson M.C., Tisch R., Tsokos G.C. (2014). IL-2 protects lupus-prone mice from multiple end-organ damage by limiting CD4-CD8- IL-17-producing T cells. J. Immunol..

[60] Humrich J.Y., von Spee-Mayer C., Siegert E., Alexander T., Hiepe F., Radbruch A., Burmester G.R., Riemekasten G. (2015). Rapid induction of clinical remission by low-dose interleukin-2 in a patient with refractory SLE. Ann. Rheum. Dis..

[61] von Spee-Mayer C., Siegert E., Abdirama D., Rose A., Klaus A., Alexander T., Enghard P., Sawitzki B., Hiepe F., Radbruch A. (2016). Low-dose interleukin-2 selectively corrects regulatory T cell defects in patients with systemic lupus erythematosus. Ann. Rheum. Dis..

[62] Zhao C., Chu Y., Liang Z., Zhang B., Wang X., Jing X., Hao M., Wang Y., An J., Zhang X. (2019). Low dose of IL-2 combined with rapamycin restores and maintains the long-term balance of Th17/Treg cells in refractory SLE patients. BMC Immunol..

[63] He J., Zhang X., Wei Y., Sun X., Chen Y., Deng J., Jin Y., Gan Y., Hu X., Jia R. (2016). Low-dose interleukin-2 treatment selectively modulates CD4(+) T cell subsets in patients with systemic lupus erythematosus. Nat. Med..

[64] He J., Zhang R., Shao M., Zhao X., Miao M., Chen J., Liu J., Zhang X., Zhang X., Jin Y. (2020). Efficacy and safety of low-dose IL-2 in the treatment of systemic lupus erythematosus: A randomised, double-blind, placebo-controlled trial. Ann. Rheum. Dis..

[65] Odler B., Windpessl M., Krall M., Steiner M., Riedl R., Hebesberger C., Ursli M., Zitt E., Lhotta K., Antlanger M. (2021). The Risk of Severe Infections Following Rituximab Administration in Patients with Autoimmune Kidney Diseases: Austrian ABCDE Registry Analysis. Front. Immunol..

[66] Tummala R., Abreu G., Pineda L., Michaels M.A., Kalyani R.N., Furie R.A., Morand E.F. (2021). Safety profile of anifrolumab in patients with active SLE: An integrated analysis of phase II and III trials. Lupus Sci. Med..

[67] Kaul A., Gordon C., Crow M.K., Touma Z., Urowitz M.B., van Vollenhoven R., Ruiz-Irastorza G., Hughes G. (2016). Systemic lupus erythematosus. Nat. Rev. Dis. Primers.

